# Editorial: Anti-cancer drug delivery: lipid-based nanoparticles

**DOI:** 10.3389/fonc.2023.1248272

**Published:** 2023-07-20

**Authors:** Alaaldin M. Alkilany, Abdelbary Elhissi, Walhan Alshaer, Amit Kunwar, Jyotsnendu Giri

**Affiliations:** ^1^ College of Pharmacy, QU Health, Qatar University, Doha, Qatar; ^2^ Cell Therapy Center, The University of Jordan, Amman, Jordan; ^3^ Radiation and Photochemistry Division, Bhabha Atomic Research Centre, Mumbai, India; ^4^ Department of Biomedical Engineering, Indian Institute of Technology Hyderabad, Kandi, India

**Keywords:** lipid-based nanoparticles, nanotherapeutics, drug delivery systems, liposomal formulations, cancer

Cancer continues to pose significant challenges that require extensive attention and efforts from the scientific community. The battle against cancer encompasses the development of effective and safe therapeutic approaches. However, achieving this balance is highly complex for anticancer therapies, as they often exhibit intense intrinsic cytotoxicity, affecting both cancerous and healthy cells and resulting in substantial toxicity that limits their clinical utility. A promising strategy to address this challenge involves the selective guidance of therapeutic agents to the cancer site, minimizing off-target effects. Nanotechnology offers powerful tools to engineer smart and targeted therapeutics that preferentially accumulate in cancerous tissues (1). This preferential localization is achieved through the Enhanced Permeation and Retention (EPR) effect, first reported by Prof. Hiroshi Maeda in 1984 (2). The EPR effect leverages the leaky vasculature in tumor regions, enabling enhanced infiltration of nanotherapeutics and localizing their therapeutic effects, which is commonly described as “passive targeting “. On the other hand, nanotechnologists may also employ “active targeting” by modifying nanoparticle surfaces with homing ligands that selectively recognize cancer cells (3). Both passive and active targeting strategies are keys for success of nanoparticle-based drug delivery systems, and serve as a justification for the development of nanotherapeutics. Extensive literature exists on various types of nanoparticles and nanomaterials with potential applications as drug delivery systems.

This Research Topic specifically focuses on lipid-based nanoparticles, which have emerged as one of the most extensively utilized nanotechnology platforms in drug delivery (4). Lipid nanoparticles was the first nanotherapeutic to secure FDA approval of Doxil® in 1995 (**Figure 1**) which is a delivery system for the anti-cancer drug doxorubicin (5). Recently, lipid-based nanoparticles have played a pivotal role in combating the global COVID-19 pandemic by facilitating the delivery of mRNA vaccines (6, 7). The successful clinical implementation of lipid-based nanoparticles in this context underscores their significant and growing presence in the field of drug delivery (**Figure 1**).

**Figure 1:**
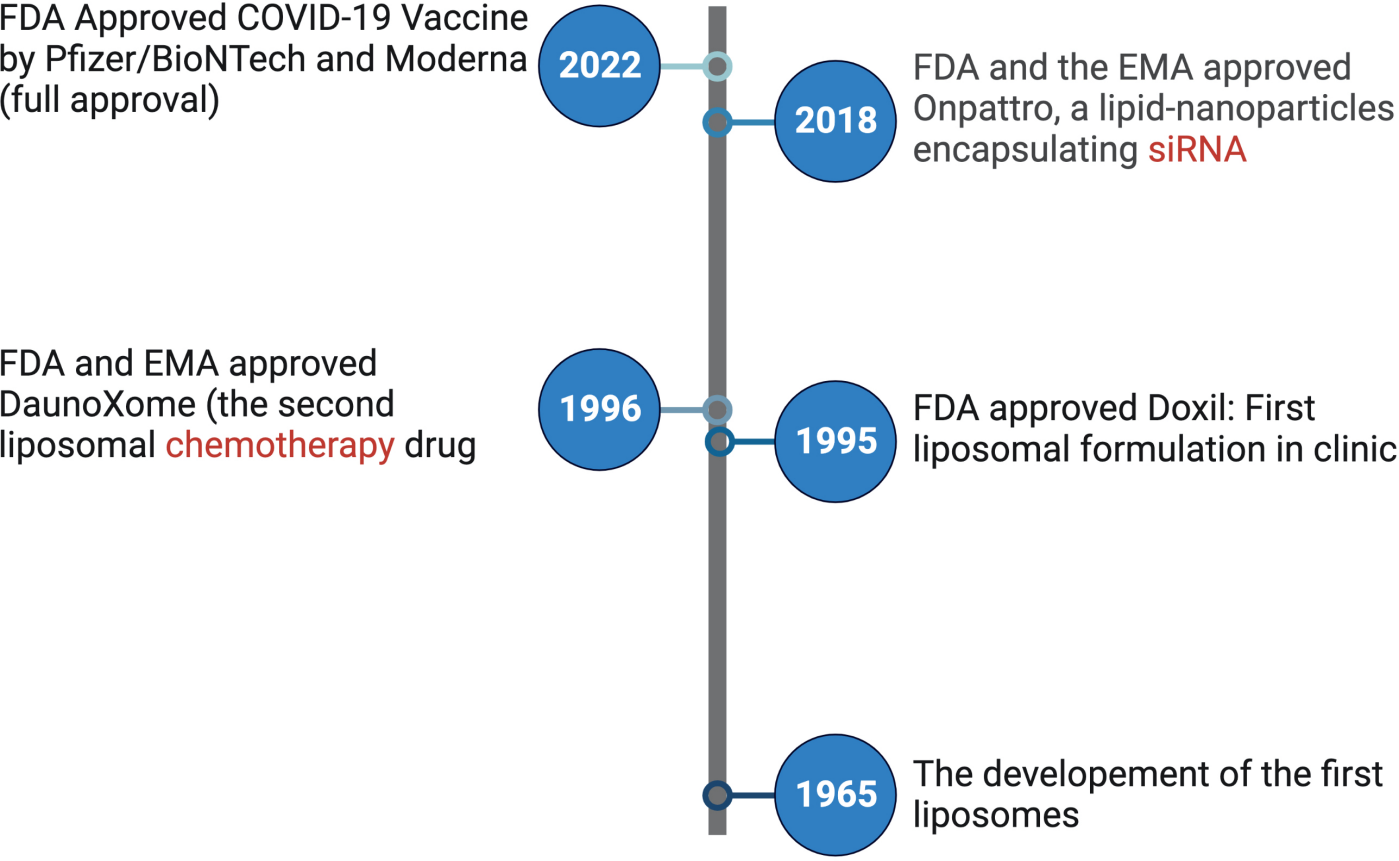
Timeline of selected important milestones for lipid nanoparticle-based therapeutic development as labeled. LNP, lipid nanoparticles; siRNA, small interfering RNA; FDA, United States Food and Drug Administration; EMA, European Medicines Agency.

Our Research Topic combines both clinical and preclinical evaluations of lipid-based nanoparticles and presents crucial findings that contribute to our collective understanding of lipidic nanoparticles in human, animal models, and cell cultures. Zhou et al. investigated the pharmacokinetics (PK) and safety profile of paclitaxel liposomes in patients with non-small cell lung cancer using population PK models. Their three-compartment model accurately described PK as well as exposure-safety relationship of paclitaxel liposome. The most important observation of this study was the probable association of neutropenia with higher exposure of paclitaxel liposomes. This is an invaluable source of information for clinicians for optimizing the clinical dosage of paclitaxel liposomes. Li et al. evaluated the bioequivalence and safety of generic and brand pegylated liposomal doxorubicin in breast cancer patients. Their multicenter crossover study confirmed the bioequivalence and comparable safety of the generic liposomal formulation to the reference product (Caelyx®). These results promote the momentum of generic nanotherapeutics which should ultimately widen the use of nanomedicine in the clinic.

Liver inflammation is associated with hepatocellular carcinoma (HCC) development; triiodothyronine (T3), being an anti-inflammatory drug may inhibit the hepatocarcinogenesis. Sun et al. reported the effective inhibition of hepatocarcinogenesis using T3-loaded liposomes *via* regulating the Inflammatory Microenvironment. This observation was confirmed *in vitro* and in rat model and the molecular mechanism was explored and reported. The highlight of the study was the evidence showing selective absorption of lipo-T3 by hepatic macrophases and remarkable reduction of drug associated side effects. Arsiwala et al. examined how low-intensity focused ultrasound (LiFUS)-mediated disruption of the blood-tumor barrier (BTB) affects the outcomes of survival of mice with brain metastases from triple-negative breast cancer (TNBC) when combined with a pegylated liposomal nanotherapeutics that circulates in the body for an extended period. Their results indicate that the combination of LiFUS and Doxil significantly increased the survival and slowed tumor progression in mice with TNBC brain metastases due to prolonged activity of doxorubicin within the tumor lesions. Finally, Hegde et al. provided the therapeutic applications of lipid-based nanoplatforms in central nervous system (CNS) tumors, with a focus on brain targeting, imaging, and immunotherapy. There critical analysis of the available literature concluded that Lipid-based nanoplatforms hold promise for precise and effective treatment of CNS tumors, with potential for revolutionizing cancer therapy through enhanced drug delivery and immunotherapy

## Author contributions

All authors listed have made a substantial, direct, and intellectual contribution to the work and approved it for publication.
